# Supplementary figures and images for: Developmental Plasticity Is Bound by Pluripotency and the Fgf and Wnt Signaling Pathways
Source: Cell Rep. 2012 Oct 25;2(4):756–65. doi: 10.1016/j.celrep.2012.08.029 (PMC3607220; doi:10.1016/j.celrep.2012.08.029)

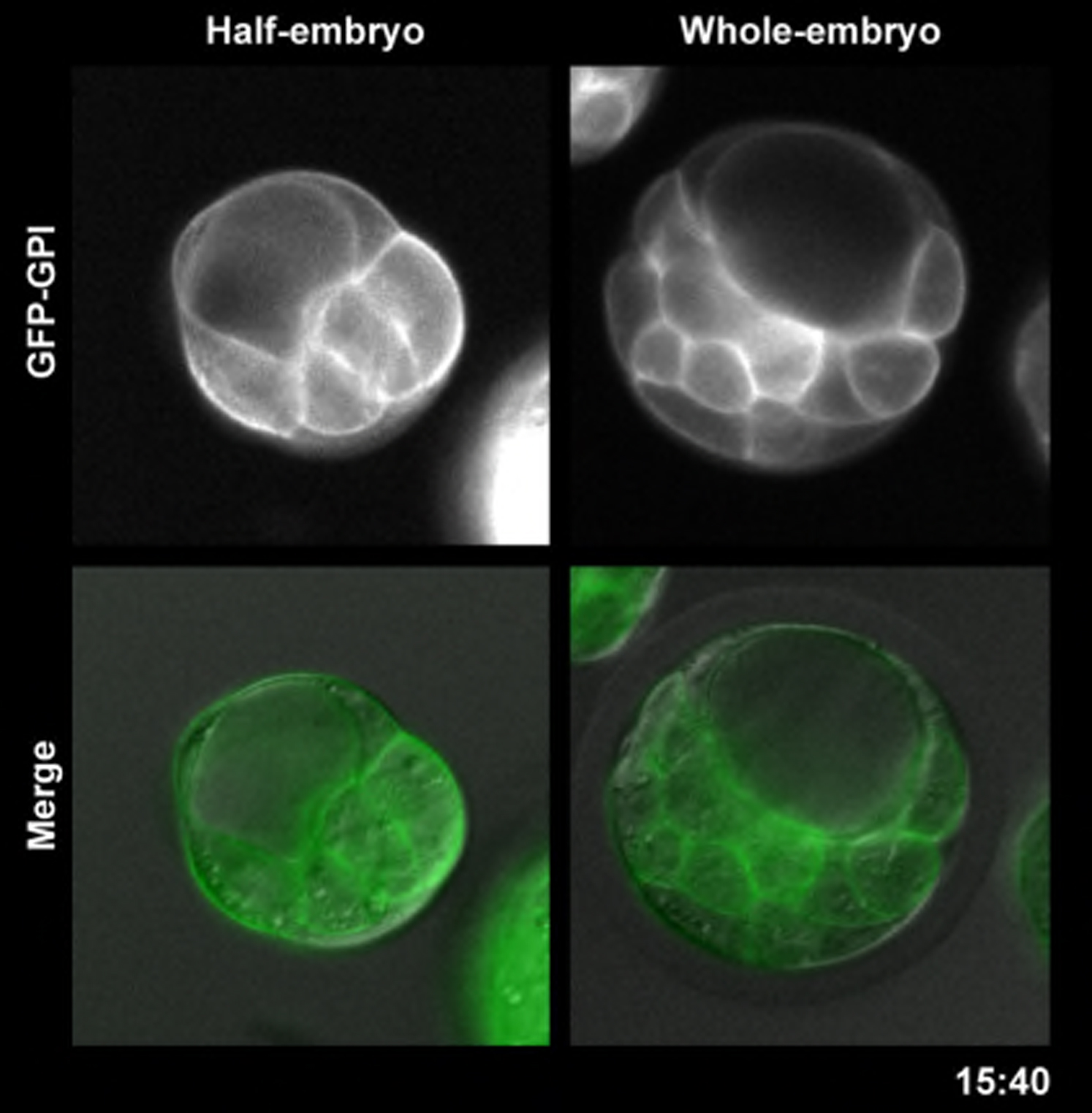

Supplement: Movie S1. Time-Lapse Imaging of Half and Whole Embryos, Related to Figure 1 — Movie of GFP:GPI transgenic half and whole embryos imaged in the same culture drop from the four(eight)-cell stage until the late-blastocyst stage. [file mmc1.jpg]

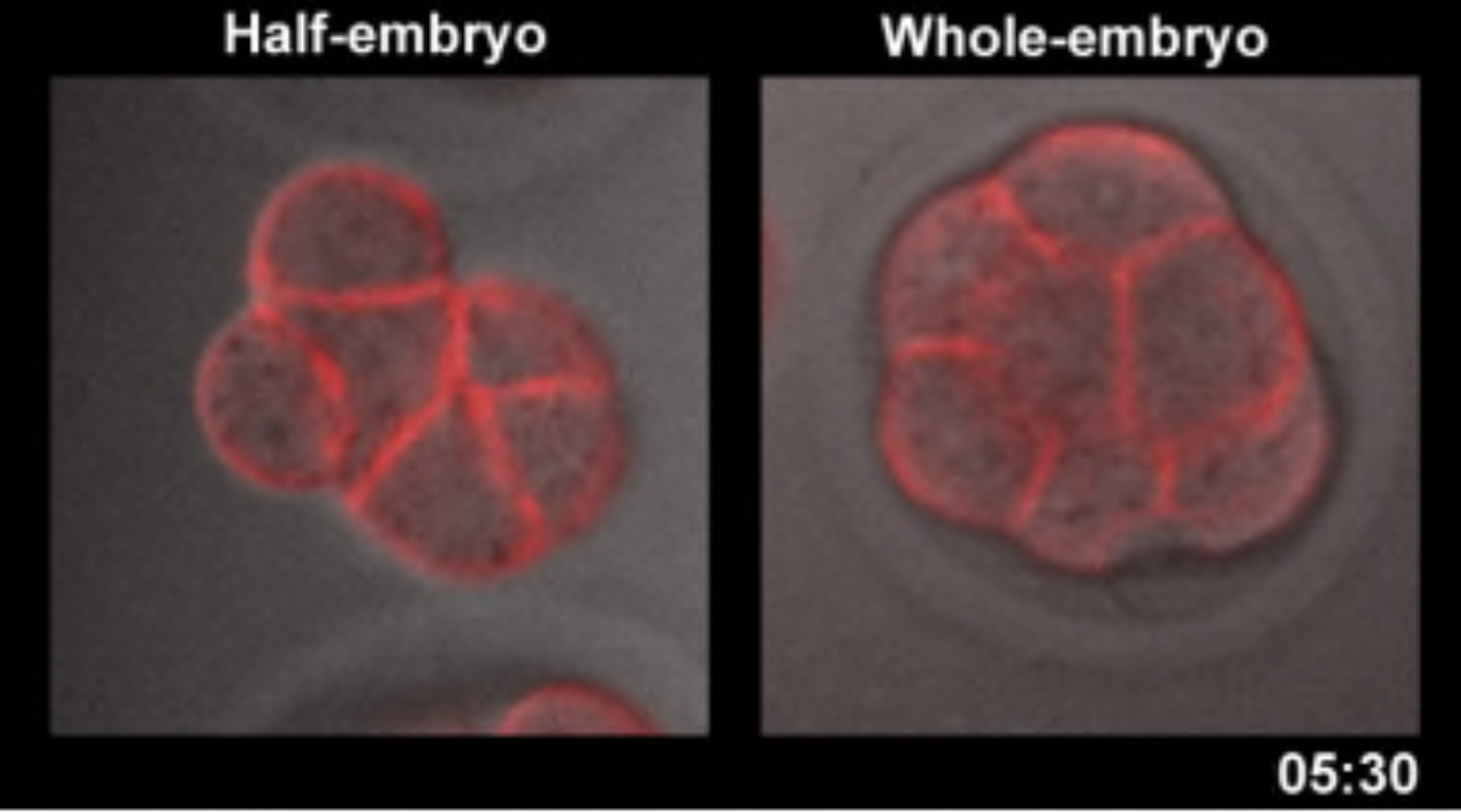

Supplement: Movie S2. Developmental Schedule of Half and Whole Embryos, Related to Figure 1 — Movie of GAP43-RFP-injected half and whole embryos to visualize cell membranes, imaged in the same culture drop. Blastomeres compact with very similar timing. [file mmc2.jpg]

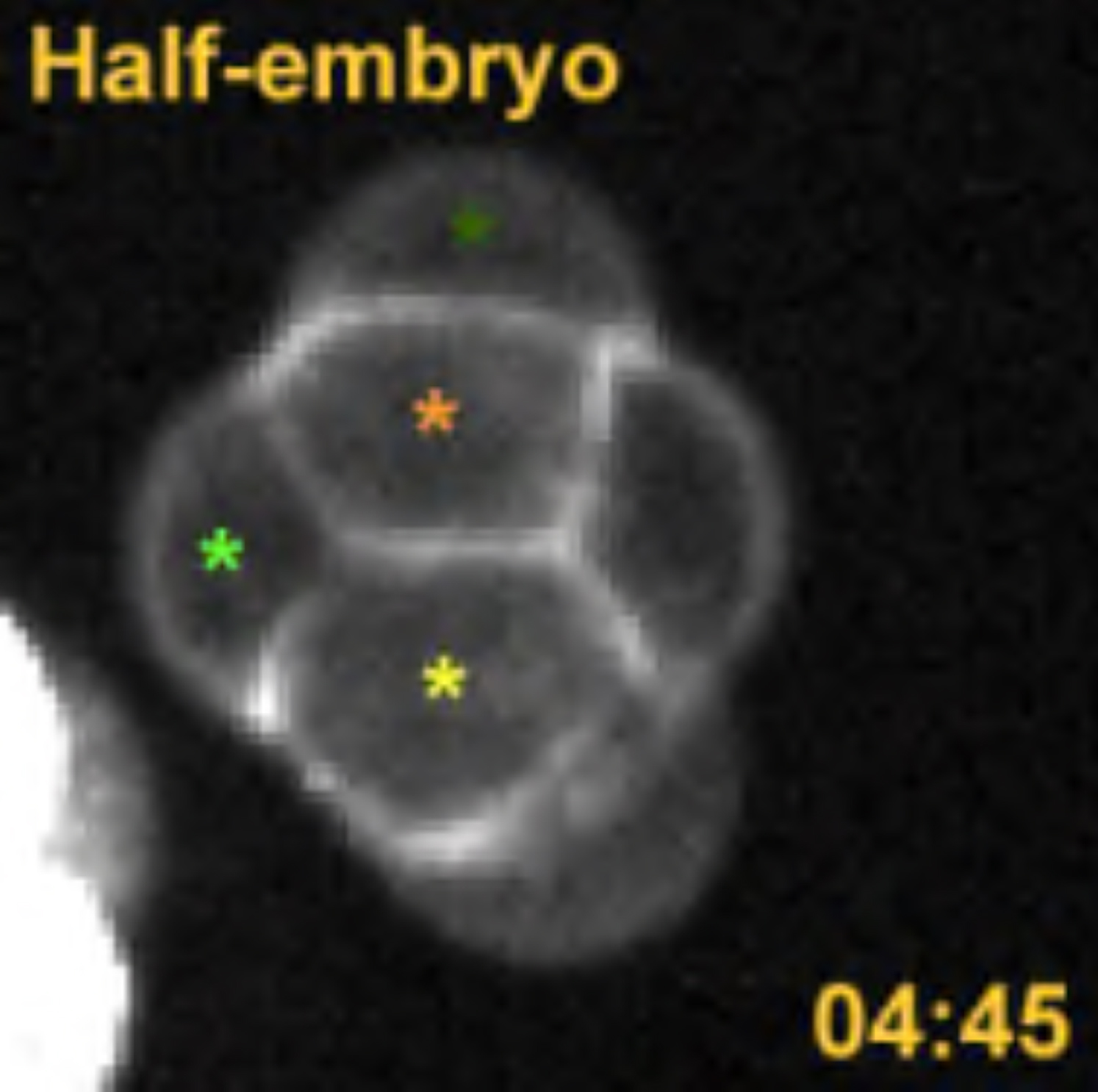

Supplement: Movie S3. Cell Internalization by Engulfment in Half Embryos, Related to Figure 1 — High-resolution time-lapse confocal imaging of a half embryo injected with GAP43-RFP to visualize cell membranes. Frames from this movie are shown in Figure 1D. Imaging begins at the transition from the 4(8)- to 8(16)-cell stage. The blastomeres marked with red dashed lines divide to generate daughters with outside domains. Daughters marked in yellow and orange are engulfed. Frames from this movie are shown in Figure 1D. Time: hours:minutes. [file mmc3.jpg]

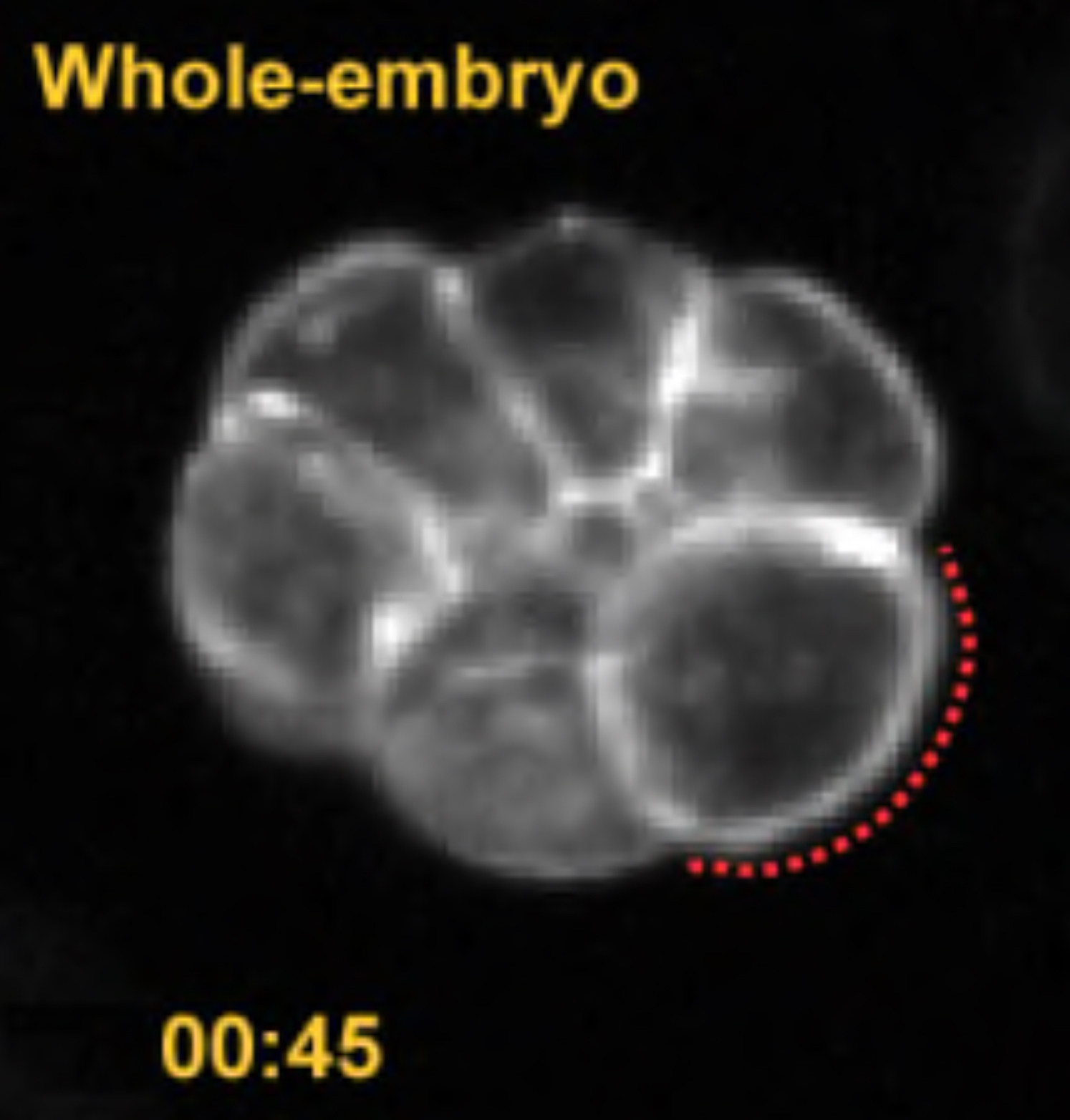

Supplement: Movie S4. Cell Internalization by Asymmetric Division in Whole Embryos, Related to Figure 1 — High-resolution time-lapse confocal imaging of a whole embryo injected with GAP43-RFP to visualize cell membranes. Imaging begins at the transition from the 8- to 16-cell stage. The blastomere marked with a red dashed line divides asymmetrically to generate inside and outside daughters. Frames from this movie are shown in Figure 1D. Time: hours:minutes. [file mmc4.jpg]
