# Supplementary material for: Developmental Plasticity Is Bound by Pluripotency and the Fgf and Wnt Signaling Pathways
Source: Cell Rep. 2012 Oct 25;2(4):756–65. doi: 10.1016/j.celrep.2012.08.029 (PMC3607220; doi:10.1016/j.celrep.2012.08.029)
Supplement: Document S1. Article plus Supplemental Information [file mmc5.pdf]

Cell PRESS
Open ACCESS
Cell Reports
Report

Developmental Plasticity Is Bound by Pluripotency and the Fgf and Wnt Signaling Pathways

Samantha A. Morris,[1,2,3] Yu Guo,[1,2] and Magdalena Zernicka-Goetz[1,2,*]
[1]Wellcome Trust/Cancer Research Gurdon Institute, Cambridge CB2 1QN, UK
[2]Department of Physiology, Development and Neuroscience, University of Cambridge, Downing Street, Cambridge CB2 3DY, UK
[3]Present address: Department of Biological Chemistry and Molecular Pharmacology, Harvard Medical School, and Boston Children's Hospital, Boston, MA 02115, USA
*Correspondence: mzg@mole.bio.cam.ac.uk
http://dx.doi.org/10.1016/j.celrep.2012.08.029

SUMMARY

Plasticity is a well-known feature of mammalian development, and yet very little is known about its underlying mechanism. Here, we establish a model system to examine the extent and limitations of developmental plasticity in living mouse embryos. We show that halved embryos follow the same strict clock of developmental transitions as intact embryos, but their potential is not equal. We have determined that unless a minimum of four pluripotent cells is established before implantation, development will arrest. This failure can be rescued by modulating Fgf and Wnt signaling to enhance pluripotent cell number, allowing the generation of monozygotic twins, which is an otherwise rare phenomenon. Knowledge of the minimum pluripotent-cell number required for development to birth, as well as the different potentials of blastomeres, allowed us to establish a protocol for splitting an embryo into one part that develops to adulthood and another that provides embryonic stem cells for that individual.

INTRODUCTION

One of the most distinguishing features of mammalian development is the plasticity with which embryos adapt to experimental perturbation, a process known as regulative development. Classically, following the destruction of one cell of the two-cell mouse embryo, the remaining cell can often compensate and support development to term (Nicholas and Hall, 1942; Tarkowski, 1959). This plasticity is preserved at later developmental stages, at least in some cases. This is because although cells separated from the four- or eight-cell mouse embryo cannot develop beyond implantation (Rossant, 1976; Tarkowski and Wróblewska, 1967), they can contribute to all tissues in chimeras (Kelly, 1977; Piotrowska-Nitsche and Zernicka-Goetz, 2005). In agreement with this, when cells are repositioned, development often readjusts (Rossant and Lis, 1979). Even chimeras built of embryos placed together can regulate to generate only one individual (Mintz, 1964; Tarkowski, 1961). This plasticity might indicate that early mammalian development, in contrast to development of other species, is stochastic. Yet there are indications in earlier work that this developmental plasticity might not be as universally applicable as generally assumed. For example, many blastomeres separated at the two-cell stage do not succeed in development to birth, and despite many efforts, production of monozygotic twins through this route has been practically unattainable (Papaioannou and Ebert, 1995; Tsunoda and McLaren, 1983). Neither the reasons behind this limitation nor the mechanism underlying developmental plasticity are currently understood.

The objective to be attained before implantation is to produce a blastocyst that has the three cell types required for subsequent development: the epiblast (EPI), which provides pluripotent cells (the foundation for the future body), and the primitive endoderm (PE) and trophectoderm (TE), extraembryonic tissues that are essential for embryo patterning and development of the placenta to ensure contact between the embryo and the mother (Zernicka-Goetz et al., 2009). The TE comprises the outer layer of cells of the blastocyst, whereas the EPI and PE correspond respectively to the deep and surface layers of the inner cell mass (ICM). These inside cells are generated in the fourth and fifth cleavage divisions, typically through differentiative divisions (Johnson and Ziomek, 1981; Pedersen et al., 1986; Morris et al., 2010), although cell engulfment was recently reported as an alternative route (McDole et al., 2011; Yamanaka et al., 2010). The first inside cells to be generated give rise predominantly to EPI, and the next set of inside cells to be generated gives birth predominantly to PE (Morris et al., 2010). These two cell types initially can be mixed, but they subsequently sort into the two layers by the mature blastocyst stage (Chazaud et al., 2006; Meilhac et al., 2009; Morris et al., 2010; Plusa et al., 2008).

Here, we investigate why some embryos regulate their development successfully, whereas in other cases development fails. Our study determines the minimum number of pluripotent cells that are essential for successful development to birth, and shows that the potential of individual blastomeres to provide this number differs. This allowed us to devise a protocol for splitting an embryo into two parts: one that develops to birth and one that provides an embryonic stem cell (ESC) line for that individual.

Cell PRESS
Open ACCESS

RESULTS AND DISCUSSION

Developmental Clock in Regulative Development

To gain insight into the mechanisms that endow mammalian embryos with developmental flexibility, and the reasons for its limitations, we first split two-cell mouse embryos into halves and followed their developmental dynamics in detail, in parallel with normal-sized embryos (Figure 1). We used high-resolution, long-term, four-dimensional (4D), live-embryo imaging to investigate with spatial and temporal precision (1) the timing of all cell divisions, (2) the division orientations of all cells, (3) the spatial arrangements of cells relative to each other, (4) the direction of cell movement, (5) the frequency of apoptosis (if it occurred), and (6) the formation of the three distinct lineages at the mature blastocyst stage. To visualize all cells continuously throughout these first 3 days of development with precision, we studied embryos expressing a glycosylphosphatidylinositol-tagged GFP membrane marker (GFP-GPI). Cellular dynamics were tracked manually with the help of SimiBiocell software (Bischoff et al., 2008).

Our spatial and temporal analyses of half-embryo developmental dynamics revealed that the processes of cell compaction, polarization, generation of inside cells, blastocyst cavity formation, and lineage allocation all occurred at the same time as in whole embryos, albeit with half the number of total cells (n = 81; Figures 1A–1C and S1A; Movies S1 and S2). Surprisingly, we found that despite the severely reduced cell number, some ICM cells of half embryos also underwent apoptosis, initiated with exactly the same timing as in full-sized embryos. Thus, key preimplantation events in half embryos follow a "clock" that runs at the same pace as in full-size embryos, in agreement with earlier observations (Le Douarin and McLaren, 1984), although, interestingly, the mechanisms of some of these developmental transitions differ (see further below).

Generation of Distinct Cell Lineages in the Course of Regulative Development

In normal development, the allocation of cells to the ICM typically occurs through differentiative, or asymmetric, cell divisions in which outside cells divide in an orientation that directs one daughter inward (Johnson and Ziomek, 1981). In contrast, both daughters of conservative, or symmetric, divisions remain outside and will contribute to TE. During whole-embryo development, there are two major waves of such cell internalization, at the 8- to 16-cell and 16- to 32-cell transitions, both of which give rise to equal proportions of ICM (Bischoff et al., 2008; Morris et al., 2010; Pedersen et al., 1986). Surprisingly, by following cell division orientations and cell positioning, we observed that the great majority of the ICM (90%; n = 81 cells, 9 embryos; p = <0.001, t-test) in half embryos was generated in the first wave (now the 4- to 8-cell transition), with the second wave generating only 10% of ICM (Figure 1D).

Because the proportion of ICM generated in the first and second waves differed drastically between half and whole embryos, we examined the process of cell internalization in greater detail. To follow this process with the highest possible resolution, we injected synthetic mRNA for membrane-associated GAP43-RFP and imaged embryos by confocal microscopy throughout the period when inside cells are generated by a first asymmetric division wave (Figure 1E, Movie S3). Unexpectedly, we observed that when cells divided in the half embryos, each daughter cell had outside domains and subsequently one daughter became engulfed. This accounted for the great majority of cell internalizations in half embryos (93%, n = 18). In contrast, we were able to detect cell engulfment in whole embryos in only 14% of cases (n = 18; Figures 1E and S1D; Movie S4).

Considering that experimental disruption of cell polarity was reported to lead to similar cell engulfment in whole embryos (Plusa et al., 2005), we wished to determine whether differences in apical polarization between cells could account for the observed engulfment. To that end, we examined the apical polarization of half-embryo cells at the four-cell stage (i.e., developmentally at the eight-cell stage). Our results showed that all exposed surfaces of cells were polarized, as assessed by apical aPKCζ staining. However, immediately following cell division, 20% (8/40) of outside cells in half embryos were devoid of aPKCζ, which was never observed in outside cells of whole embryos (0/26; Figure 1F). Such unpolarized cells would be expected to be engulfed by more polarized neighbors (Plusa et al., 2005). This may also explain why cell engulfment was recently observed after injection of eight-cell-stage blastomeres, because such an injection could disrupt cell polarization (Morris, 2011; Yamanaka et al., 2010).

To confirm the engulfment of cells with reduced apical polarity, we filmed cell divisions of embryos injected at the two-cell stage with E-cadherin-GFP mRNA (Figure 1G). We observed that in whole embryos, asymmetric divisions led inside rather than outside cells to inherit basally localized E-cadherin-GFP (100%, n = 8/8 dividing cells). In contrast, in half embryos, E-cadherin was inherited by outside cells and, importantly, cells that inherited higher levels of E-cadherin were subsequently engulfed (75%, n = 6/8 dividing cells; Figure 1G).

Relationship between Cell Fate and the Timing of Cell Internalization

We previously reported that the first inside cells to be generated in undisturbed development are biased to contribute to the EPI, whereas those that arrive inside later are biased to differentiate to the extraembryonic PE (Morris et al., 2010). To determine whether a similar relationship between the timing of cell origin and fate allocation is displayed during regulative development, we carried out 4D lineage analyses of all cells. This revealed that the first inside cells in half embryos contributed equally to EPI and PE lineages (n = 71 cells). In contrast, inside cells that arose later were significantly biased toward differentiating into PE (90%, $p < 0.001$, n = 10 cells, t-test; Figure 1H), similar to what was observed during undisturbed development.

In our next analyses we aimed to determine whether half-embryos regulating their development show a relationship between cell origin (wave 1 versus wave 2) on the one hand, and cell position, apoptosis and final cell fate on the other, as reported in normal development (Morris et al., 2010). Tracking of all cell behaviors revealed that when the blastocyst formed, the wave-2-derived inner cells were positioned at the ICM surface and consequently gave rise to PE. Thus, the position of these cells was "correct" in relation to their subsequent fate, and indeed we did not observe these cells to change their

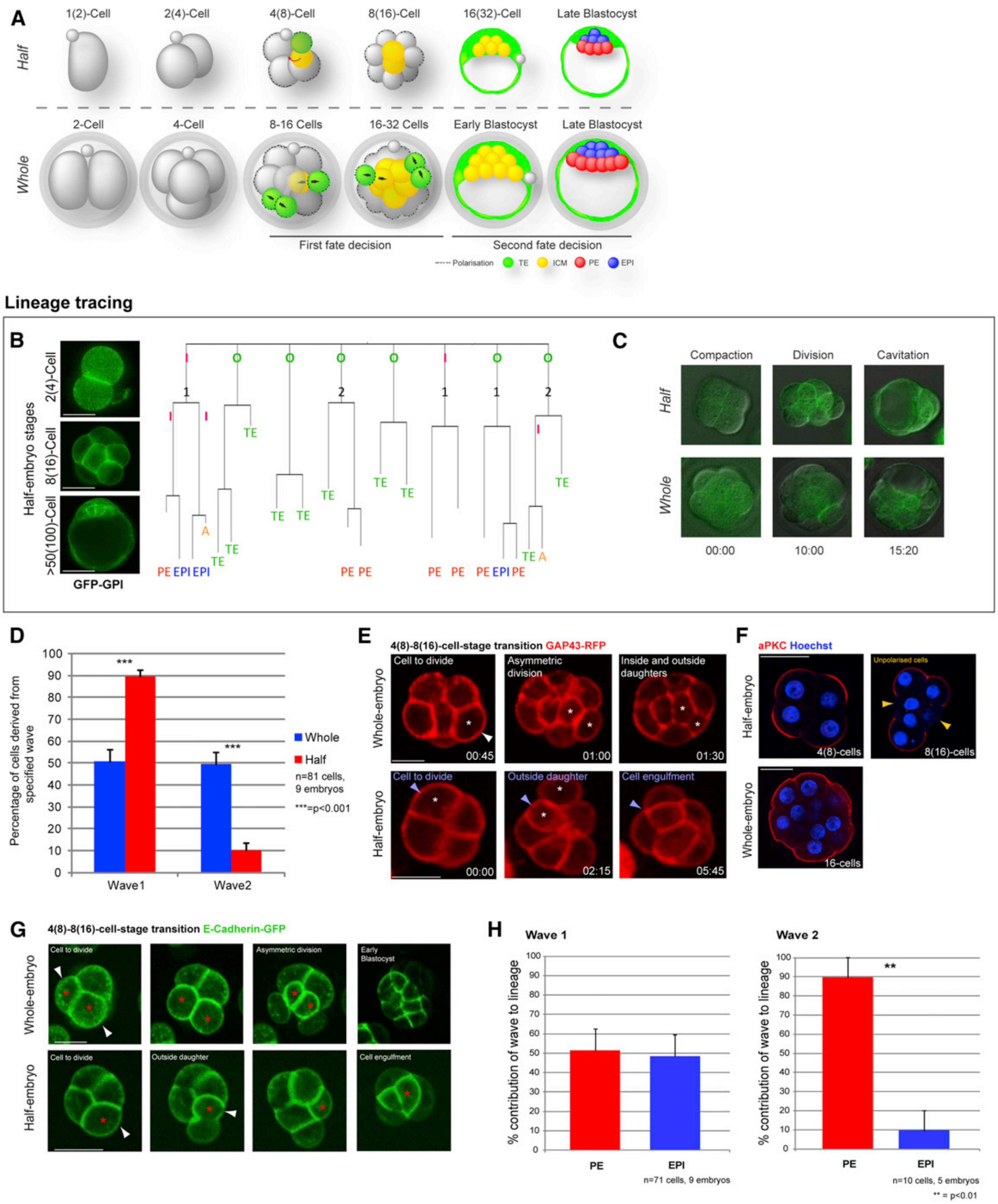

Figure 1. Half-Embryo Development

(A) Developmental progression of half embryos (upper row, equivalent stages in parentheses) and whole embryos (lower row).

(B) Time-lapse imaging of half-embryo development. Lineage diagram: A, apoptosis; I, inside cell; O, outside cell; 1, wave 1 internalization; 2, wave 2 internalization.

(C) Compaction, division, and cavitation occur with the same timing in half and whole embryos imaged side by side (merged differential interference contrast and GFP frames from Movie S1).

(D) Proportions of first- and second-wave internalizations in whole embryos (from Morris et al., 2010) and half embryos.

(E) Time-lapse imaging of cell internalization by asymmetric division (whole embryo) and engulfment (half embryos). Time: hours:minutes.

(F) aPKCζ immunostaining of half and whole embryos in the 4(8)- to 8(16)-cell-stage transition. Scale bars: 50 μM (B) and 25 μM (D and E).

(G) Time-lapse imaging of cell internalization by asymmetric division (whole embryo) and engulfment (half embryos) in embryos expressing E-cadherin-GFP.

(H) Final fates of cells derived from the first and second waves in half embryos.

All error bars indicate standard error. See also Figure S1 and Movies S2, S3, and S4.

position and move into the deeper, EPI compartment. However, we found that wave 1 cells could either be in a correct position (i.e., deep within the ICM) or in an incorrect position (i.e., at the surface) in relation to their future fate (Figure S1B). Interestingly, our data revealed the highest incidence of apoptosis in cells that were positioned incorrectly (Figure S1B). Moreover, despite the lower cell content in the half embryos, the timed onset of apoptosis was the same as in undisturbed development (Figure S1C). These findings indicate that the mechanisms used by half embryos to form a blastocyst are similar to those employed by their undisturbed counterparts, including apoptosis despite low cell numbers. However, the exception is cell internalization, which is driven nearly entirely by cell engulfment rather than differentiative division, possibly due to geometrical constraints.

Relationship between Pluripotent Cell Number and Successful Development

Our detailed examination of each developmental transition suggested that half embryos show high variability in ICM cell number. To confirm this, we determined the precise numbers of pluripotent (Nanog-positive), PE (Sox17-positive), and TE (Cdx2-positive) cells at the end of preimplantation development. We found that in contrast to undisturbed development, EPI cell number in half embryos varied drastically, from 0 to 8 cells, in comparison with 8–12 cells in full-sized embryos (Figure 2A). This EPI cell number correlated with PE cell number, reflecting the total ICM size, whereas the TE proportion was relatively constant (Figure S2).

It was previously demonstrated that although all blastomeres at the four-cell stage are pluripotent, they differ significantly in developmental potential depending on the cleavage division pattern by which they arise (Piotrowska-Nitsche et al., 2005; Piotrowska-Nitsche and Zernicka-Goetz, 2005; Torres-Padilla et al., 2007). We therefore wondered whether half embryos with fewer ICM cells might be derived from blastomeres with reduced potential. To test this hypothesis, we monitored division orientation and order at the two- to four-cell-stage transition to identify and isolate individual blastomeres known to differ in potential (Figure 2B). This allowed us to construct half-embryo chimeras from two four-cell blastomeres (i.e., two animal-vegetal [AV] parts [AV-AV]) that were previously shown to have full developmental potential (Piotrowska-Nitsche et al., 2005; see Experimental Procedures and legend to Figure 2B), and half-embryo chimeras from blastomeres of restricted potential (two vegetal blastomeres [V-V]). When we compared their development, we found that in contrast to the AV-AV half-embryo chimeras, the V-V chimeras failed to form a substantial ICM, suggesting that the developmental history of blastomeres can influence successful development. In agreement with this, when we generated chimeras in which V-blastomeres were paired with AV- or A-blastomeres, cell allocation to the EPI was enhanced ($p < 0.01$, t-test; Figure 2B). On initial consideration, the larger EPI population observed in A-V chimeras was unexpected, considering the reduced potential of their component blastomeres. It can be reasoned, however, that the high Cdx2 expression and cell polarization discovered in V-blastomere eight-cell stage progeny (Jedrusik et al., 2008) may lead to the engulfment of A-blastomere progeny, thus promoting ICM generation.

Because our experiments demonstrated a variable EPI cell number in half embryos, it was important to determine whether this correlates with embryo viability. Thus, we grouped embryos according to EPI cell number using cell position as the criterion for cell identity (deep cells: EPI; surface cells: PE; Morris et al., 2010) and transferred them into foster mothers together with whole-sized-embryo carriers of different genotype, handled under the same conditions. We first recovered embryos 2 days after implantation and correlated EPI cell number with developmental success (Figure 2C). This revealed that a minimum of four EPI cells was required for reliable development to embryonic day 6.5 (E6.5), demonstrating that development beyond implantation correlates with the number of EPI cells attained by implantation.

To confirm and extend these observations, we next sought to determine whether EPI cell number correlates with successful development to birth. To that end, we placed half embryos into groups depending on whether they had low-EPI cell numbers (0–2 EPI cells) or high-EPI cell numbers (4–5 EPI cells) together with whole-embryo carriers. Only two live births resulted from the transfer of 9 low-EPI half embryos (22%, n = 2/9), whereas almost all (87%, n = 7/8) high-EPI half embryos developed to term. Two weeks after birth, the half-embryo-derived pups were comparable in size to their whole-embryo littermates (Figure 2D). Together with our lineage analysis, these results indicate that insufficient allocation of cells to the EPI by the time of implantation presents a barrier to successful development. Our results also indicate that cell internalization, apoptosis, and the developmental history of the cells all influence the size of this population.

Expanding the Pluripotent Cell Population Promotes Development

We argued that if indeed EPI cell number is the primary limiting factor for successful development, then half-embryo development should be rescued by expanding the EPI population. It was reported that small-molecule inhibitors of extracellular signal-related kinase (ERK1/2) and glycogen synthase kinase 3β (GSK3) (2i; Ying et al., 2008) prevent PE formation and expand EPI (Nichols et al., 2009). We therefore treated half embryos with 2i from four-cell to late-blastocyst stages. This resulted in a 30% increase in the total ICM cell number ($p < 0.05$, t-test, n = 36 embryos; Figure 3A). However, after 2i treatment, all inner cells developed as EPI. Because this would prevent development due to the absence of PE (Chazaud et al., 2006), we next incubated half embryos in 2i only until the mid-blastocyst stage and then switched to inhibitor-free medium to permit PE formation (Nichols et al., 2009). This regimen increased the EPI compartment from, on average, three cells to eight cells ($p < 0.01$, t-test) while maintaining PE integrity (Figure 3B). Because this transient 2i treatment increased EPI cell number to a level critical for development to birth, we transferred such half embryos to foster mothers. We found that development of the 2i-treated half embryos was significantly rescued, doubling the frequency of egg cylinder formation (63%, n = 18) relative to untreated half embryos (33%, n = 16; Figure 3C).

We next addressed whether such a supplement of pluripotent cells would increase the frequency of monozygotic twinning,

Cell PRESS
Open ACCESS

A

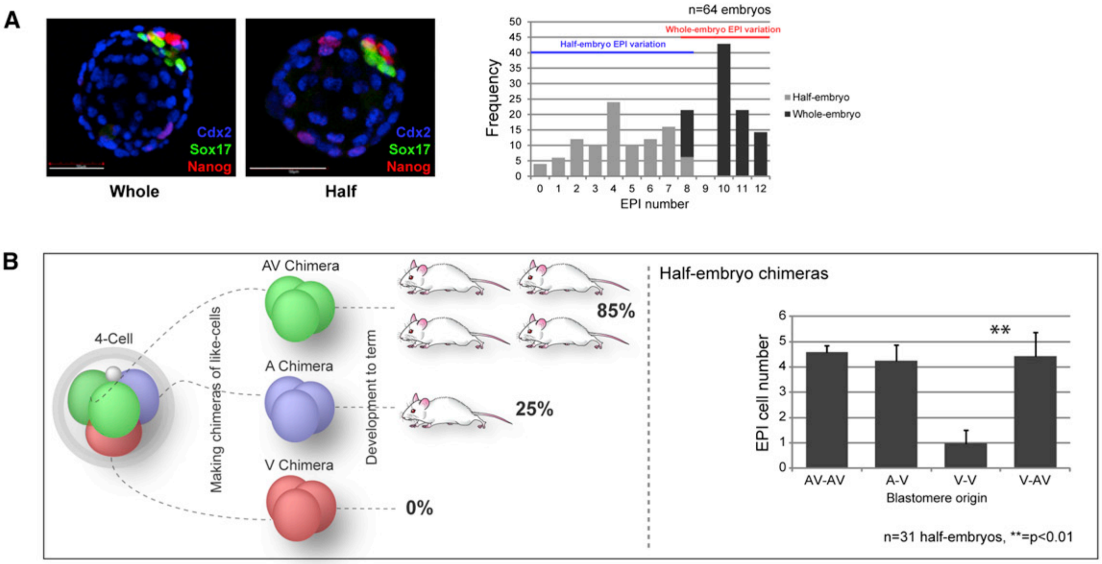

C

n = 71 half-embryos and 60 carriers transferred to 16 recipients

	Transferred		Recovered		% Half-embryo recovery
	Half-embryos	*Carriers*	*Half-embryos*	*Carriers*	(E6.5, normalised to carriers)
0 EPI	13	12	2 (15%)	9 (75%)	21%
1 EPI	15	11	3 (20%)	9 (82%)	24%
2 EPI	7	7	1 (14%)	5 (71%)	20%
3 EPI	10	8	1 (10%)	7 (88%)	11%
4 EPI	10	9	5 (50%)	7 (78%)	64%
5 EPI	16	13	9 (56%)	9 (69%)	81%

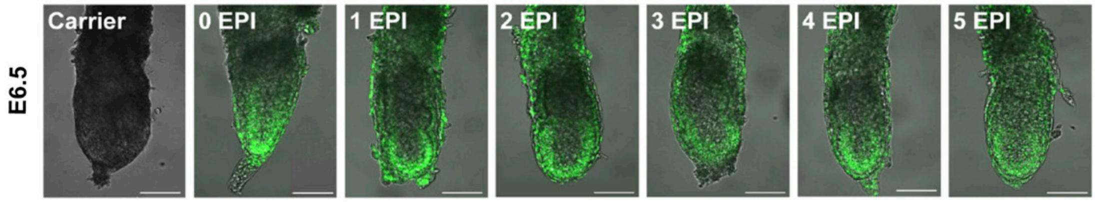

D

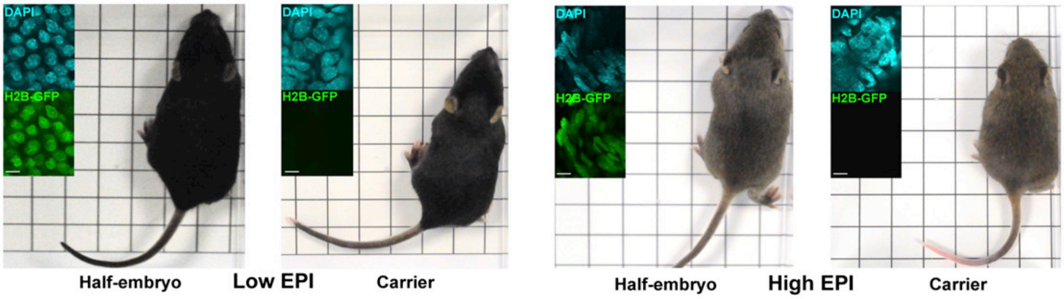

	Low EPI halves	Carrier embryos
Embryos transferred	9	8
Live births (P14)	2 (22%)	6 (75%)
Average weight (P14)	13.80g	11.90g

	High EPI halves	Carrier embryos
Embryos transferred	8	7
Live births (P14)	7 (87.5%)	6 (86%)
Average weight (P14)	12.35g	10.10g

Figure 2. ICM Size and Embryo Viability

(A) Nanog (EPI), Sox17 (PE), and Cdx2 (TE) immunostaining of whole and half embryos. Bar chart: EPI number frequency in half embryos.

which was previously shown to be very inefficient when splitting embryos at the two-cell stage (Tsunoda and McLaren, 1983). To assess twinning, we separated cells at the two-cell stage and performed the 2i rescue treatment as described above before transferring the twin half-blastocysts to foster mothers. We found that this doubled the frequency of singletons and twins recovered in comparison with 2i-untreated control twins (two singletons and three pairs of twins versus two singletons and one pair of twins, respectively; $p < 0.05$; Figure 3D). This provides further support for the notion that developmental success relies on adequate generation of a pluripotent cell population, and that regulative development can be enhanced by treatments that expand the pluripotent domain.

The Degree of 2i-Mediated Rescue Reflects the Developmental Potential of Four-Cell Blastomeres

Because we were able to identify a protocol that would increase the efficiency of twinning, we considered whether it might also be possible to rescue the development of embryos generated from individual four-cell-stage blastomeres (referred to as quarter embryos), which are known to form trophoblastic vesicles and die after implantation (Rossant, 1976; Tarkowski and Wróblewska, 1967). To determine this, we dissociated cells of the four-cell embryo and then exposed individual blastomeres transiently to 2i, followed by culture to the blastocyst stage, as for half-size embryos. We found that these 2i-treated quarter embryos had as much as 3-fold more EPI cells (as assessed by Nanog expression) compared with quartered control embryos (n = 30 cells, $p < 0.01$, t-test; Figure 3E). However, the number of EPI cells in these 2i-treated quarter embryos did not reach the minimum threshold of four, and upon transfer to fosters, no embryos developed to E6.5 (0/20 untreated, 0/20 treated, three experiments; data not shown).

We observed that the quarter embryos differed in their ability to generate pluripotent cells (6/14, 43%, with evident ICM), even after 2i treatment through to E4.5 to maximize EPI, at the expense of PE. We wondered whether this could be explained by the known different developmental histories/origins of the blastomeres. To address this, we monitored division patterns at the two- to four-cell transition, as before, to identify individual four-cell blastomeres, and then cultured them to the blastocyst stage. This revealed that only embryos derived from AV blastomeres reliably generated pluripotent cells (Figure 3F), in agreement with our findings in half embryos. Together, these results indicate that blastomeres at the four-cell stage differ in their potential to regulate development.

Establishing ES Cell Lines while Maintaining Embryo Viability

Knowing the developmental properties of different embryo fragments led us to explore whether it might be possible to split an embryo into two parts—one that could develop fully and one that could reliably generate a pluripotent cell line for that individual. Considering that quarter-embryo development could not be fully rescued, removing a quarter fragment did not seem appropriate to achieve this goal. Therefore, we decided to remove three blastomeres from an eight-cell embryo (this also avoids selecting only cells derived from compromised V cells). We found that blastocysts developed from these three blastomeres possessed an average of three EPI cells (Figure 4A). In contrast, the remaining five blastomeres developed into blastocysts containing more than five EPI cells (Figure 4A), which, as we show here, should be a sufficient number for development to birth. To check whether this is indeed the case, we transferred the 5/8th part embryos to foster mothers and found that they developed to term, similarly to whole embryos (six out of eight 5/8th embryos versus five out of six whole embryos developed to give P14 pups; Figure 4F). In contrast, the 3/8th blastocysts did not develop following their transfer (n = 13, data not shown).

To determine whether the 3/8th blastocysts could provide a source of stem cells, we stimulated their inner cell allocation using 2i, followed by ESC derivation (n = 14/17, 82%; Figures 4B, S3A, and S3B; Extended Experimental Procedures). This allowed us to establish six independent ESC lines with an overall efficiency of 82% (Figures 4C, S3, and S4) that contributed to all germ layers, demonstrating their pluripotency (Figures 4D, 4E, and S5). These results indicate that three eight-cell blastomeres provide the minimum developmental unit required to reliably establish pluripotent ESCs but not embryo viability.

Conclusions

Our results indicate that for regulative development to be successful, a critical minimum number of four pluripotent cells must be generated before implantation, and this can be compromised by the strict developmental clock of cell polarization, cell internalization, and blastocyst formation. This limitation can be overcome by increasing the number of pluripotent cells by modulating Fgf and Wnt signaling. Although blastomeres separated at the two-cell stage can be rescued by this treatment, blastomeres separated at the four-cell stage cannot. Knowing the precise number of pluripotent cells required for development to birth, and that not all blastomeres have equal potential enabled us to devise a regime for splitting an embryo into two parts: one that develops to birth and one that provides a source of ESCs.

EXPERIMENTAL PROCEDURES

Embryo Culture and Disaggregation

Two-cell mouse embryos were collected in M2 medium from superovulated (C57BL/6xCBA) females mated with wild-type, transgenic CAG::GFP-GPI

(B) EPI number in chimeric half embryos constructed from blastomeres with full developmental potential (AV), reduced potential (A), and restricted potential (V).
(C) Development to postimplantation stages (E6.5) of H2B-GFP transgenic half embryos grouped according to EPI number, relative to wild-type whole-embryo carriers.
(D) Development to term of H2B-GFP transgenic half embryos with low or high EPI number, relative to wild-type whole-embryo carriers.
Scale bars: 50 μM (A), 100 μM (C), and 10 μM (D, inset); 1 cm^2 grid (D). All error bars indicate standard error. See also Figure S2.

Cell PRESS
Open ACCESS

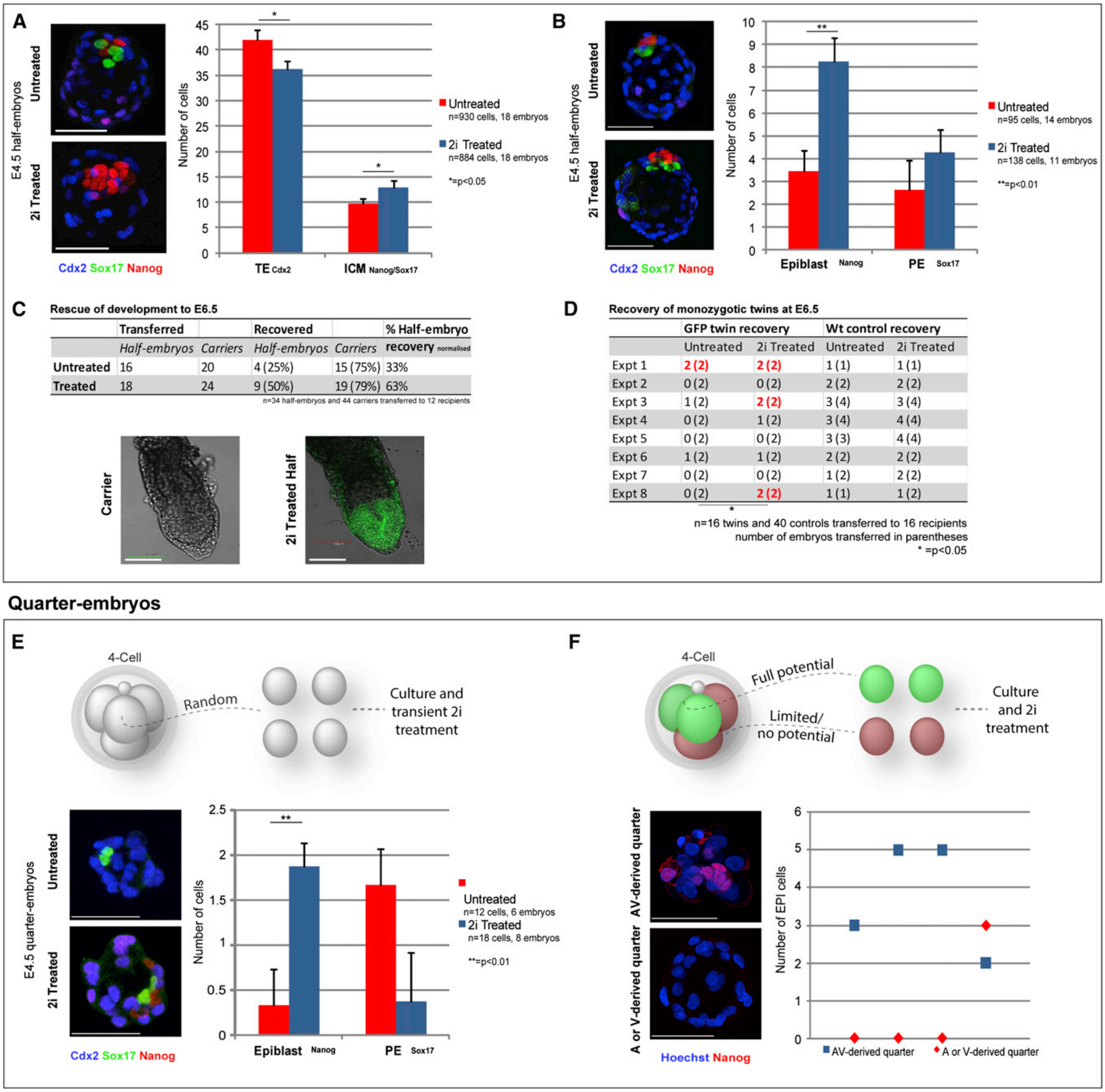

	Transferred		Recovered		% Half-embryo
	Half-embryos	Carriers	Half-embryos	Carriers	recovery normalised
Untreated	16	20	4 (25%)	15 (75%)	33%
Treated	18	24	9 (50%)	19 (79%)	63%

	GFP twin recovery		Wt control recovery	
	Untreated	2i Treated	Untreated	2i Treated
Expt 1	2 (2)	2 (2)	1 (1)	1 (1)
Expt 2	0 (2)	0 (2)	2 (2)	2 (2)
Expt 3	1 (2)	2 (2)	3 (4)	3 (4)
Expt 4	0 (2)	1 (2)	3 (4)	4 (4)
Expt 5	0 (2)	0 (2)	3 (3)	4 (4)
Expt 6	1 (2)	1 (2)	2 (2)	2 (2)
Expt 7	0 (2)	0 (2)	1 (2)	2 (2)
Expt 8	0 (2)	2 (2)	1 (1)	1 (2)

Figure 3. Rescue of Half-Embryo Development

(A) Nanog, Sox17, and Cdx2 immunostaining (projection) of 2i-treated (from four-cell stage to late blastocyst) and untreated half embryos: ICM and TE numbers.

(B) Nanog, Sox17, and Cdx2 immunostaining (projection) of transiently 2i-treated and untreated half embryos: EPI and PE numbers.

(C) Development to postimplantation stages (E6.5) of 2i-treated and untreated H2B-GFP transgenic half embryos, relative to wild-type whole-embryo carriers.

(D) Development to postimplantation stages (E6.5) of 2i-treated and untreated H2B-GFP transgenic twins (*p < 0.05, chi-square test).

(E) Attempted rescue of quarter-embryo development. Nanog, Sox17, and Cdx2 immunostaining (projection) of 2i-treated and untreated half embryos: EPI and PE numbers.

(F) ICM size (Nanog immunostaining, projection) in 2i-treated quarter embryos with known full developmental potential (AV-derived), and restricted potential (A- or V-derived).

Scale bars: 50 μM (A), 100 μM (B), and 50 μM (C and D). All error bars indicate standard error.

(Rhee et al., 2006) or H2B-GFP (Hadjantonakis and Papaioannou, 2004) males. Half and quarter embryos were generated following zona pellucida removal, with acidic Tyrode's solution and pipetting to separate blastomeres. Embryos were cultured in KSOM medium in 5% CO_2 as previously described (Morris et al., 2010). For half- and quarter-embryo rescues, KSOM was supplemented with 2i (1 μM PD0325901 and 3 μM Chiron; Cayman Chemical).

Cell PRESS
Open ACCESS

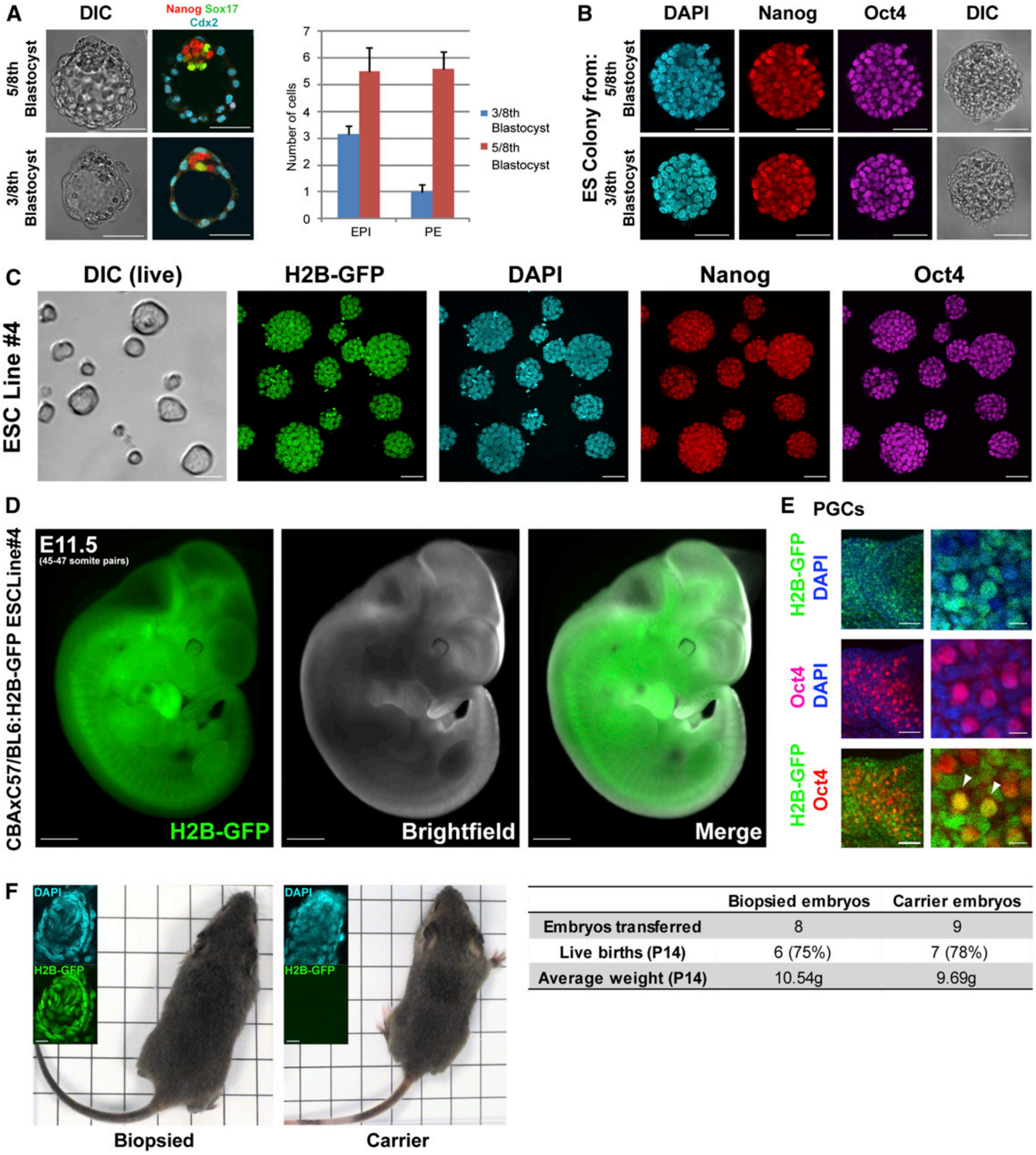

	Biopsied embryos	Carrier embryos
Embryos transferred	8	9
Live births (P14)	6 (75%)	7 (78%)
Average weight (P14)	10.54g	9.69g

Figure 4. Generation of ESCs and Mice from Single Embryos

(A) Nanog, Sox17, and Cdx2 immunostaining of 5/8th and 3/8th blastocysts. Bar chart: EPI and PE numbers.

(B) Nanog and Oct4 immunostaining (single slice) of ES colonies derived from whole embryos and 3/8th blastocysts by immunosurgery, followed by culture in 2i+LIF. Nuclei were counterstained with DAPI.

(C) Nanog and Oct4 immunostaining (single slice) of ESC line 4, derived from H2B-GFP transgenic 3/8th embryos. Nuclei were counterstained with DAPI.

(D) Fluorescent and bright-field images of a chimera of a wild-type CBAxC57/BL6 embryo and H2B-GFP transgenic ESC line 4.

(E) Oct4 immunostaining of genital ridges from the embryo in (D) to visualize primordial germ cells derived from ESCs (white arrows). Nuclei were counterstained with DAPI.

(F) Comparison of 2-week-old pups derived from 5/8th H2B-GFP embryos and wild-type whole-embryo littermates.

Scale bars: 50 μM (A–C), 1 mm (D), 50 μM (E, left panels), 10 μM (E, right panels), 10 μM (F, inset); 1 cm^2 grid (F). All error bars indicate standard error. See also Figures S3, S4, and S5.

Time-Lapse Imaging

Green-fluorescence and transmitted-light multisection images were acquired every 15 min with a Hamamatsu ORCA ER CCD camera on 15 focal planes every 4 μm, with an exposure of 4 ms for transmitted light and 200 ms for fluorescence for 3 days. SimiBiocell software was used for cell tracking (Bischoff et al., 2008). An Intelligent Imaging Solutions Spinning Disk confocal microscope was used to image cell internalization by acquiring red-fluorescence and transmitted-light multisection images.

Microinjection with mRNAs for GAP43-RFP or E-cadherin-GFP at a concentration of 400 ng/μl, synthesized from the pRN3P construct, was carried out as previously described (Zernicka-Goetz et al., 1997).

Immunostaining was carried out as previously described (Torres-Padilla et al., 2007) with the following primary antibodies: goat anti-Sox17 (R&D Systems), rabbit anti-Nanog (Abcam), mouse anti-Cdx2 (BioGenex), rabbit anti-aPKCζ (Santa Cruz), and mouse anti-Oct4. Nuclei were counterstained with either DAPI or Hoechst.

Monitoring the Cleavage Pattern

Embryos were microinjected with rhodamine dextran at the two-cell stage, and division orientation was monitored every 15 min until the three- and four-cell stages. Embryos were grouped according to cleavage orientation and sequence as described by Piotrowska-Nitsche et al. (2005), followed by disaggregation and culture as quarter embryos or reaggregation into half embryos.

Development of Embryos to E6.5, E11.5, or Term

To assess development, half, quarter, biopsied 5/8th embryos and 3/8th embryos were transferred to pseudopregnant females (Weber et al., 1999). Manipulated embryos from transgenic H2B-GFP mice were transferred along with wild-type carriers to monitor the efficiency of transfer.

SUPPLEMENTAL INFORMATION

Supplemental Information includes Extended Experimental Procedures, five figures, and four movies and can be found with this article online at http://dx.doi.org/10.1016/j.celrep.2012.08.029.

LICENSING INFORMATION

This is an open-access article distributed under the terms of the Creative Commons Attribution 3.0 Unported License (CC-BY; http://creativecommons.org/licenses/by/3.0/legalcode).

ACKNOWLEDGMENTS

We thank Lewis Wolpert for the inspiration for this study; Sean Jeffries, Harry Leitch, and David Glover for discussions; Emma Rawlins for help with imaging the E11.5 embryos; and the M.Z.G. group for support. This work was supported by a Wellcome Trust grant to M.Z.G.

Received: July 9, 2012
Revised: August 15, 2012
Accepted: August 27, 2012
Published online: October 4, 2012

REFERENCES

Bischoff, M., Parfitt, D.E., and Zernicka-Goetz, M. (2008). Formation of the embryonic-abembryonic axis of the mouse blastocyst: relationships between orientation of early cleavage divisions and pattern of symmetric/asymmetric divisions. Development *135*, 953–962.

Chazaud, C., Yamanaka, Y., Pawson, T., and Rossant, J. (2006). Early lineage segregation between epiblast and primitive endoderm in mouse blastocysts through the Grb2-MAPK pathway. Dev. Cell *10*, 615–624.

Eakin, G.S., and Hadjantonakis, A.K. (2006). Production of chimeras by aggregation of embryonic stem cells with diploid or tetraploid mouse embryos. Nat. Protoc. *1*, 1145–1153.

Hadjantonakis, A.K., and Papaioannou, V.E. (2004). Dynamic in vivo imaging and cell tracking using a histone fluorescent protein fusion in mice. BMC Biotechnol. *4*, 33.

Jedrusik, A., Parfitt, D.E., Guo, G., Skamagki, M., Grabarek, J.B., Johnson, M.H., Robson, P., and Zernicka-Goetz, M. (2008). Role of Cdx2 and cell polarity in cell allocation and specification of trophectoderm and inner cell mass in the mouse embryo. Genes Dev. *22*, 2692–2706.

Johnson, M.H., and Ziomek, C.A. (1981). The foundation of two distinct cell lineages within the mouse morula. Cell *24*, 71–80.

Kelly, S.J. (1977). Studies of the developmental potential of 4- and 8-cell stage mouse blastomeres. J. Exp. Zool. *200*, 365–376.

Le Douarin, N., and McLaren, A. (1984). Chimeras in Developmental Biology (London, Orlando: Academic Press).

McDole, K., Xiong, Y., Iglesias, P.A., and Zheng, Y. (2011). Lineage mapping the pre-implantation mouse embryo by two-photon microscopy, new insights into the segregation of cell fates. Dev. Biol. *355*, 239–249.

Meilhac, S.M., Adams, R.J., Morris, S.A., Danckaert, A., Le Garrec, J.F., and Zernicka-Goetz, M. (2009). Active cell movements coupled to positional induction are involved in lineage segregation in the mouse blastocyst. Dev. Biol. *331*, 210–221.

Mintz, B. (1964). Formation of genetically mosaic mouse embryos, and early development of "lethal (T12/T12)-normal" mosaics. J. Exp. Zool. *157*, 273–292.

Morris, S.A. (2011). Cell fate in the early mouse embryo: sorting out the influence of developmental history on lineage choice. Reprod. Biomed. Online *22*, 521–524.

Morris, S.A., Teo, R.T., Li, H., Robson, P., Glover, D.M., and Zernicka-Goetz, M. (2010). Origin and formation of the first two distinct cell types of the inner cell mass in the mouse embryo. Proc. Natl. Acad. Sci. USA *107*, 6364–6369.

Nichols, J., Silva, J., Roode, M., and Smith, A. (2009). Suppression of Erk signalling promotes ground state pluripotency in the mouse embryo. Development *136*, 3215–3222.

Nicholas, J.S., and Hall, B.V. (1942). Experiments on developing rats. II. The development of isolated blastomeres and fused eggs. J. Exp. Zool. *90*, 441–459.

Papaioannou, V.E., and Ebert, K.M. (1995). Mouse half embryos: viability and allocation of cells in the blastocyst. Dev. Dyn. *203*, 393–398.

Pedersen, R.A., Wu, K., and Bałakier, H. (1986). Origin of the inner cell mass in mouse embryos: cell lineage analysis by microinjection. Dev. Biol. *117*, 581–595.

Piotrowska-Nitsche, K., and Zernicka-Goetz, M. (2005). Spatial arrangement of individual 4-cell stage blastomeres and the order in which they are generated correlate with blastocyst pattern in the mouse embryo. Mech. Dev. *122*, 487–500.

Piotrowska-Nitsche, K., Perea-Gomez, A., Haraguchi, S., and Zernicka-Goetz, M. (2005). Four-cell stage mouse blastomeres have different developmental properties. Development *132*, 479–490.

Plusa, B., Frankenberg, S., Chalmers, A., Hadjantonakis, A.K., Moore, C.A., Papalopulu, N., Papaioannou, V.E., Glover, D.M., and Zernicka-Goetz, M. (2005). Downregulation of Par3 and aPKC function directs cells towards the ICM in the preimplantation mouse embryo. J. Cell Sci. *118*, 505–515.

Plusa, B., Piliszek, A., Frankenberg, S., Artus, J., and Hadjantonakis, A.K. (2008). Distinct sequential cell behaviours direct primitive endoderm formation in the mouse blastocyst. Development *135*, 3081–3091.

Rhee, J.M., Pirity, M.K., Lackan, C.S., Long, J.Z., Kondoh, G., Takeda, J., and Hadjantonakis, A.K. (2006). In vivo imaging and differential localization of lipid-modified GFP-variant fusions in embryonic stem cells and mice. Genesis *44*, 202–218.

Rossant, J. (1976). Postimplantation development of blastomeres isolated from 4- and 8-cell mouse eggs. J. Embryol. Exp. Morphol. *36*, 283–290.

Rossant, J., and Lis, W.T. (1979). Potential of isolated mouse inner cell masses to form trophectoderm derivatives in vivo. Dev. Biol. *70*, 255–261.

Tarkowski, A.K. (1959). Experiments on the development of isolated blastomers of mouse eggs. Nature *184*, 1286–1287.

Tarkowski, A.K. (1961). Mouse chimaeras developed from fused eggs. Nature *190*, 857–860.

Tarkowski, A.K., and Wróblewska, J. (1967). Development of blastomeres of mouse eggs isolated at the 4- and 8-cell stage. J. Embryol. Exp. Morphol. *18*, 155–180.

Torres-Padilla, M.E., Parfitt, D.E., Kouzarides, T., and Zernicka-Goetz, M. (2007). Histone arginine methylation regulates pluripotency in the early mouse embryo. Nature *445*, 214–218.

Tsunoda, Y., and McLaren, A. (1983). Effect of various procedures on the viability of mouse embryos containing half the normal number of blastomeres. J. Reprod. Fertil. *69*, 315–322.

Weber, R.J., Pedersen, R.A., Wianny, F., Evans, M.J., and Zernicka-Goetz, M. (1999). Polarity of the mouse embryo is anticipated before implantation. Development *126*, 5591–5598.

Yamanaka, Y., Lanner, F., and Rossant, J. (2010). FGF signal-dependent segregation of primitive endoderm and epiblast in the mouse blastocyst. Development *137*, 715–724.

Ying, Q.L., Wray, J., Nichols, J., Batlle-Morera, L., Doble, B., Woodgett, J., Cohen, P., and Smith, A. (2008). The ground state of embryonic stem cell self-renewal. Nature *453*, 519–523.

Zernicka-Goetz, M., Pines, J., McLean Hunter, S., Dixon, J.P.C., Siemering, K.R., Haseloff, J., and Evans, M.J. (1997). Following cell fate in the living mouse embryo. Development *124*, 1133–1137.

Zernicka-Goetz, M., Morris, S.A., and Bruce, A.W. (2009). Making a firm decision: multifaceted regulation of cell fate in the early mouse embryo. Nat. Rev. Genet. *10*, 467–477.

Supplemental Information

EXTENDED EXPERIMENTAL PROCEDURES

Derivation of ES Colonies and ESC Lines

Whole, quarter, and 3/8th embryos were cultured from the 8-cell stage (or the developmental equivalent) to early blastocyst stage in KSOM+2i at 37°C, under 5% CO_2, followed by 2 days of culture in N2B27+2i+LIF under the same conditions (Nichols et al., 2009). ICM was recovered from the resulting expanded blastocysts by immunosurgery (Solter and Knowles, 1975), followed by culture on laminin (Sigma L2020, 10 μg/ml) coated tissue culture plastic in N2B27+2i+LIF until the colonies had attached and significantly increased in size (3-4 days). ES colonies were recovered by mouth pipette and disaggregated to single cells with 0.25% Trypsin (Invitrogen) and plated on laminin-coated tissue culture plastic to establish ESC lines. Lines were passaged at least 5 times before assessment of pluripotency by Nanog/Oct4 immunostaining and tissue contribution in chimeric embryos.

Chimeras

The pluripotency of H2B-GFP transgenic embryo-derived ESC lines was assessed by aggregation of small clumps of ES cells (10-15 cells) with wild-type C57BL/6xCBA embryos at the uncompacted 8-cell stage (Eakin and Hadjantonakis, 2006). These aggregation chimeras were cultured until the early blastocyst stage and then transferred to foster females. Foetuses were recovered at E11.5. Wholemount, 2-channel GFP and brightfield imaging was performed on a Zeiss inverted fluorescent dissection microscope. Germline contribution was assessed by dissection of genital ridges and immunostaining of Oct4 to visualize H2B-GFP ESC contribution to PGCs.

SUPPLEMENTAL REFERENCES

Eakin, G.S., and Hadjantonakis, A.K. (2006). Production of chimeras by aggregation of embryonic stem cells with diploid or tetraploid mouse embryos. Nat. Protoc. *1*, 1145–1153.

Nichols, J., Silva, J., Roode, M., and Smith, A. (2009). Suppression of Erk signaling promotes ground state pluripotency in the mouse embryo. Development *136*, 3215–3222.

Solter, D., and Knowles, B.B. (1975). Immunosurgery of mouse blastocyst. Proc. Natl. Acad. Sci. USA *72*, 5099–5102.

Cell PRESS
Open ACCESS

A

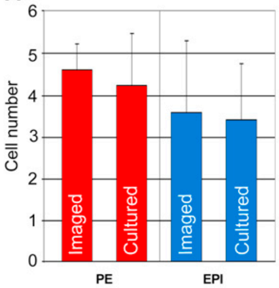

B

Cells initially deep	Total	EPI	PE	Apoptosis
Wave 1	24	17	5	2
Wave 2	2	0	1	1
Cells initially surface				
Wave 1	70	18	31	21
Wave 2	12	1	8	3

C

Half-embryo	Total ICM cells	Apoptotic Cells	% Apoptosis
1	6	3	50
2	9	4	44.4
3	8	4	50
4	12	2	16.7
5	12	3	25
6	6	0	0
7	15	1	6.7
8	16	6	37.5
9	14	0	0
10	11	4	36.4
Average	10.9	2.7	26.7

D

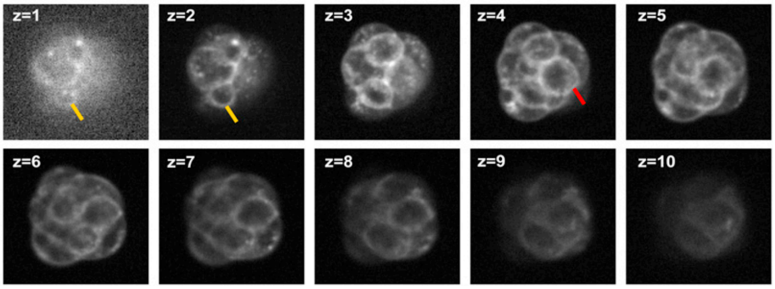

Figure S1. Time-Lapse Imaging and Lineage Tracing in Half Embryos, Related to Figure 1
(A) Nanog and Sox17 immunostaining of EPI and PE, respectively, in imaged and cultured embryos shows that imaging conditions do not adversely affect development or ICM cell sorting.
(B) Developmental origin, initial ICM position, and final fate of cells tracked in half embryos. Wave-2-derived cells initially are correctly positioned at the ICM surface. Wave-1-derived cells positioned deep are most likely to be eliminated by apoptosis, a mechanism also employed by whole embryos (Morris et al., 2010).
(C) Incidence of apoptosis in half embryos. The average is similar to that previously reported for whole embryos (Morris et al., 2010).
(D) Multiple z-slices of a GAP43-RFP-expressing half embryo at a single time point. The yellow arrow marks an outside cell in the process of engulfment, maintaining some small amount of outside contact. The red arrow marks a fully engulfed cell in the inside compartment.
All error bars indicate standard error.

Cell PRESS
Open ACCESS

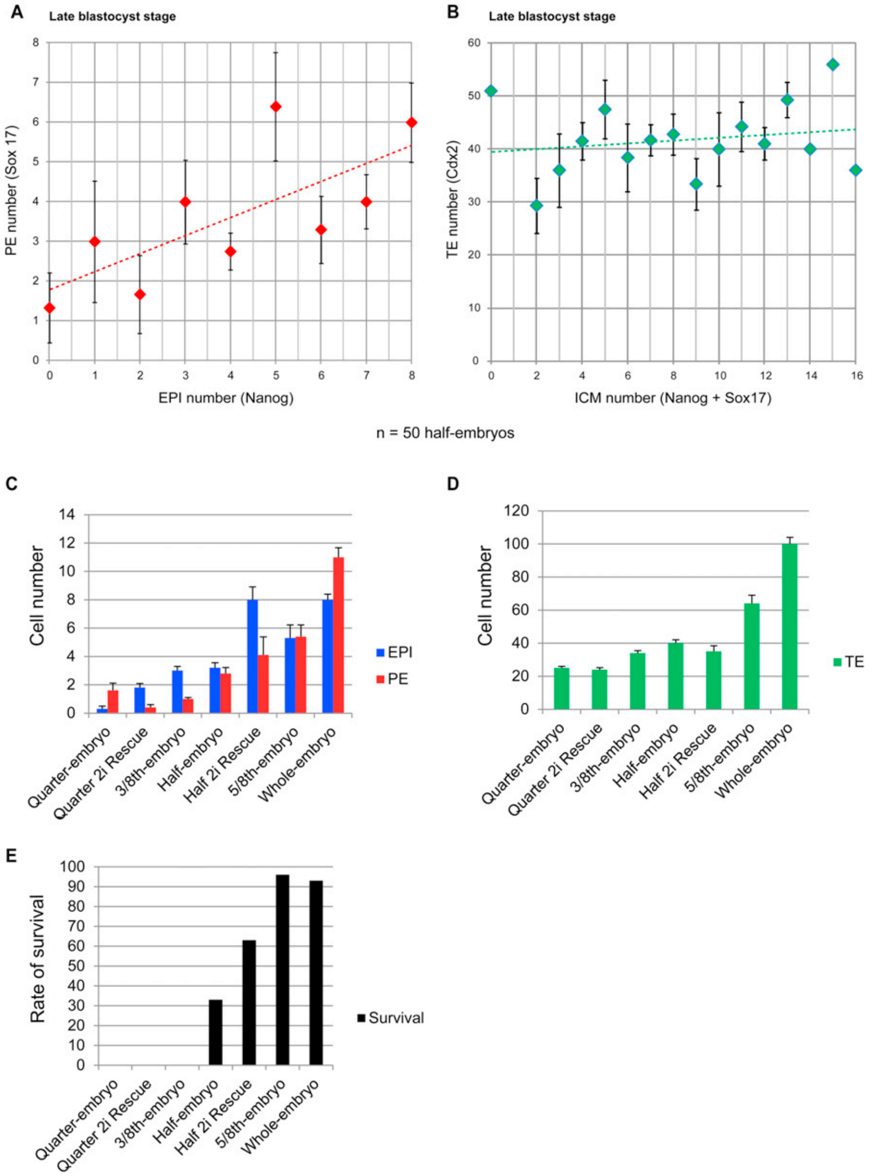

Figure S2. Half-Embryo Blastocyst Composition, Related to Figure 2

(A) Relationship between PE and EPI numbers in late-blastocyst-stage half embryos as assessed by Sox17 and Nanog immunostaining. EPI number correlates with PE number.

(B) Relationship between ICM and TE numbers in late-blastocyst-stage half embryos as assessed by Sox17, Nanog, and Cdx2 immunostaining. There is no significant correlation between ICM and TE numbers. n = 50 half embryos.

(C–E) EPI and PE numbers (C), TE number (D), and survival rates (E) of all embryo fragments and whole embryos observed in this study. The PE and TE numbers do not significantly vary between untreated and 2i-rescued half embryos, whereas EPI number significantly increases. Thus, the increased survival rate of 2i-rescued half embryos can be accounted for by the increased EPI population.

All error bars indicate standard error.

A Generation and isolation of ICM from 3/8th blastocysts

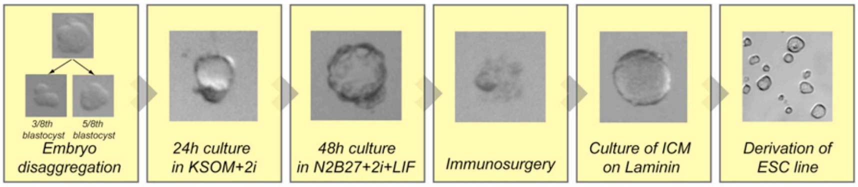

B Growth of ES colony from 3/8th blastocyst

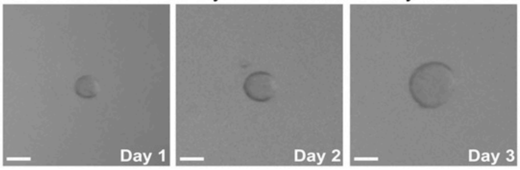

C Derivation efficiencies

Colony formation efficiencies

Whole-embryos		3/8th blastocysts		Quarter-embryos	
**Blastocysts**	ES Colonies	Blastocysts	ES Colonies	Blastocysts	ES Colonies
**8**	8 (100)	17	14 (82)	11	3 (27)

ESC derivation efficiencies

Whole-embryos		3/8th blastocysts	
ES Colonies	ESC Lines	ES Colonies	ESC Lines
3	3 (100)	6	6 (100)

Figure S3. Derivation of ESCs, Related to Figure 4

(A) Generation and isolation of ICM from embryo fragments. Eight-cell-stage embryos are split into five blastomere and three blastomere portions. The 3/8th fragment is cultured in 2i for 24 h to the early blastocyst stage, followed by a further 48 h culture in N2B27+2i+LIF. Extraembryonic tissue is removed from the 3/8th blastocyst by immunosurgery, whereupon the ICM consisting only of EPI cells is cultured on laminin in N2B27+2i+LIF for 3–5 days to form an ES colony. After sufficient growth, the colony is disaggregated with trypsin and plated on laminin to establish an ESC line. The remaining 5/8th embryo fragment is transferred to a surrogate female at the early blastocyst stage.

(B) Growth of ES colony derived from a 3/8th blastocyst over 3 days. Scale bars: 50 μM.

(C) Efficiencies of ES colony formation and ESC derivation from whole embryos, 3/8th blastocysts, and quarter embryos. Fourteen ES colonies were derived from 17 3/8th blastocysts, corresponding to 82% efficiency. Six ESC lines were established from six of these colonies, and the remaining colonies were analyzed for Nanog and Oct4 expression.

All error bars indicate standard error.

Cell PRESS
Open ACCESS

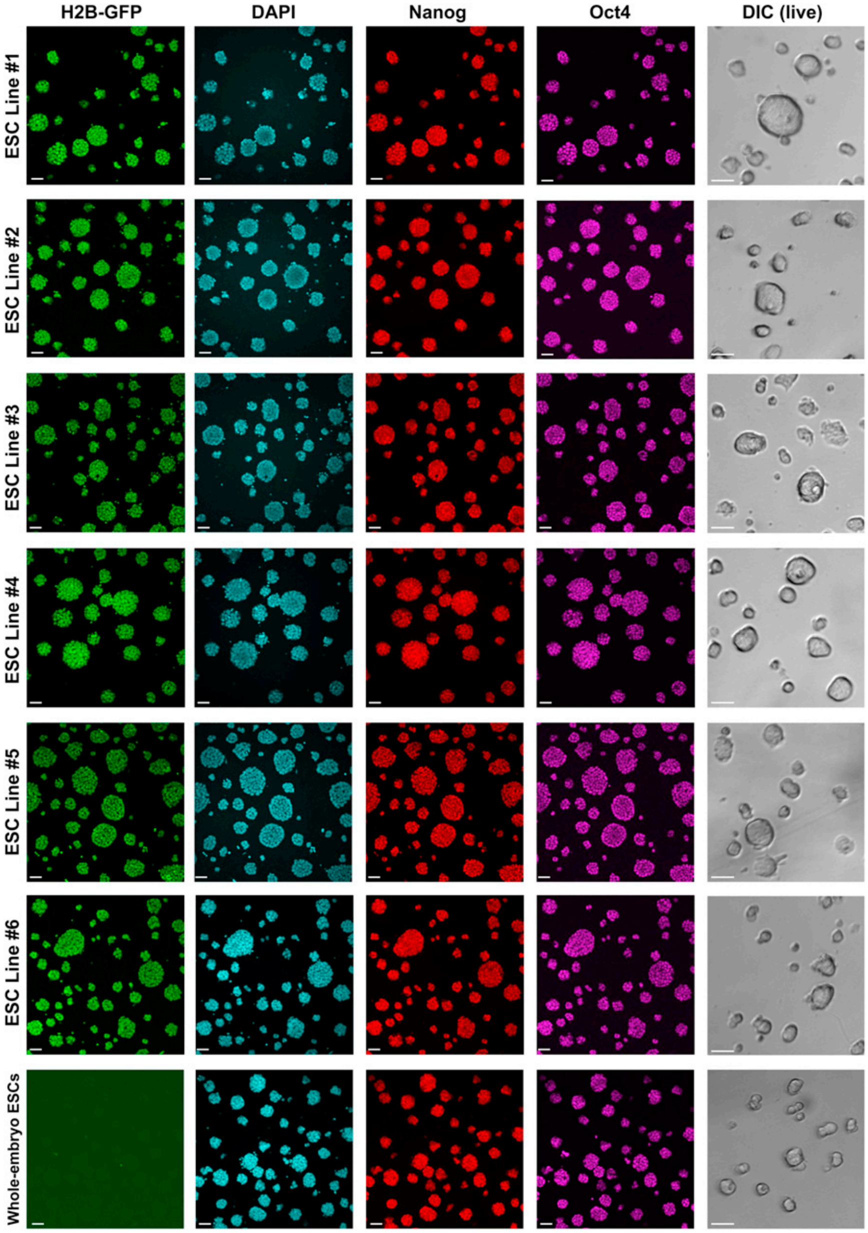

Figure S4. ESC Lines Derived from 3/8th Embryos, Related to Figure 4

All six ESC lines derived from H2B-GFP transgenic 3/8th embryos were imaged in culture (differential interference contrast) and following fixation were immunostained for Nanog and Oct4. Nuclei were counterstained with DAPI. ESCs derived from wild-type whole embryos were derived and immunostained for comparison. Scale bars: 50 μM. All error bars indicate standard error.

Cell PRESS
Open ACCESS

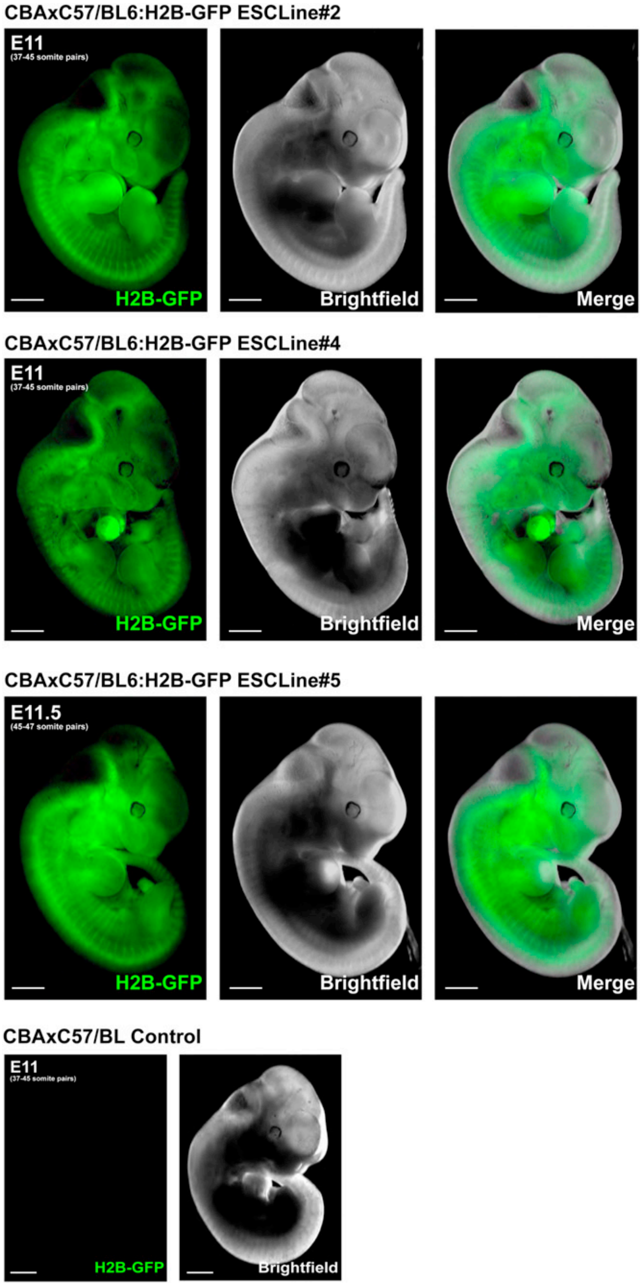

Figure S5. ESC Contribution to Chimeras, Related to Figure 4

Three of the six ESC lines derived from 3/8th embryos were aggregated with wild-type embryos at the eight-cell stage and transferred to surrogate females at the early blastocyst stage. Embryos recovered at E11.5 were imaged under fluorescence and bright field to visualize tissues derived from H2B-GFP ESC cells. Tissues from all three germ layers were extensively contributed by all three ESC lines tested. Incidence of chimerism: ESC line 2: four of four embryos chimeric; ESC line 4: four of four embryos chimeric; ESC line 5: two of three embryos chimeric. A wild-type embryo is included for comparison. Scale bars: 1 mm. All error bars indicate standard error.
